# Diagnostic accuracy of the rapid Xpert HIV-1 Viral Load XC, Xpert HIV-1 Viral Load & m-PIMA HIV-1/2 Viral Load in South African clinics

**DOI:** 10.1097/QAI.0000000000003037

**Published:** 2022-06-13

**Authors:** Jienchi DORWARD, Jessica NAIDOO, Pravikrishnen MOODLEY, Yukteshwar SOOKRAJH, Natasha SAMSUNDER, Fathima SAYED, Nivashnee NAICKER, Thomas FANSHAWE, Paul K DRAIN, Richard J LESSELLS, Gail HAYWARD, Christopher C BUTLER, Nigel GARRETT

**Affiliations:** 1 Nuffield Department of Primary Care Health Sciences, University of Oxford, Oxford, Oxfordshire, United Kingdom; 2 Centre for the AIDS Programme of Research in South Africa (CAPRISA), University of KwaZulu–Natal, Durban, KwaZulu-Natal, South Africa; 3 Department of Virology, University of KwaZulu-Natal and National Health Laboratory Service, Inkosi Albert Luthuli Central Hospital, KwaZulu-Natal, South Africa; 4 eThekwini Municipality Health Unit, eThekwini Municipality, Durban KwaZulu-Natal, South Africa; 5 Department of Global Health, Schools of Medicine and Public Health, University of Washington, Seattle, USA; 6 Department of Medicine, School of Medicine, University of Washington, Seattle, USA; 7 Department of Epidemiology, School of Public Health, University of Washington, Seattle, USA; 8 KwaZulu-Natal Research and Innovation Sequencing Platform (KRISP), University of KwaZulu-Natal, Durban, South Africa; 9 Discipline of Public Health Medicine, School of Nursing and Public Health, University of KwaZulu-Natal, Durban, KwaZulu-Natal, South Africa

**Keywords:** HIV, point-of-care, viral load testing

## Abstract

**Background:**

We aimed to evaluate the analytic performance of three rapid HIV viral load assays: the novel Xpert HIV-1 VL XC (Xpert XC), the Xpert HIV-1 VL (Xpert VL), and the m-PIMA HIV-1/2 VL (m-PIMA).

**Setting:**

Two South African clinics.

**Methods:**

We conducted a prospective diagnostic accuracy study. Site-laboratory technicians and nurses used the Xpert XC, Xpert VL and m-PIMA to test plasma samples from people with HIV receiving antiretroviral therapy. We compared results with the Roche Cobas HIV-1 reference assay. We determined accuracy to detect viraemia at the World Health Organization (WHO) failure threshold of 1000 copies/mL on all three assays, and 50 and 200 copies/mL on the Xpert assays. We assessed agreement using Bland-Altman plots.

**Results:**

We enrolled 140 participants (98 [70%] women, median age 37 years), who provided 189 paired samples at one or more timepoints. We tested 174 samples with the Xpert XC, 188 with the Xpert VL, and 128 with the m-PIMA. At 1000 copies/mL, sensitivity and specificity (95% confidence intervals) were 97% (82-100) and 98% (93-99) (Xpert XC), 100% (87-100) and 96% (91-98) (Xpert VL), and 92% (72-99) and 99% (93-100) (m-PIMA) respectively. At 50 copies/mL, sensitivity and specificity were 93% (81-98) and 96% (91-99) (Xpert XC), and 95% (84-99) and 95% (90-98) (Xpert VL) respectively. Mean bias was -0.10 (-0.54 to 0.34) log_10_ copies/mL (Xpert XC), 0.07 (-0.37 to 0.52) log_10_ copies/mL (Xpert VL) and -0.26 (-0.83 to 0.31) log_10_ copies/mL (m-PIMA).

**Conclusions:**

In these South African clinics, the accuracy of all three assays was clinically acceptable to detect viraemia at the WHO failure threshold, while both Xpert assays were also accurate at detecting low level viraemia.

## Introduction

The World Health Organization (WHO) recommends antiretroviral therapy (ART) for all people living with HIV, to suppress the HIV viral load.^1^ This prevents immunosuppression and related morbidity and mortality,^2,3^ as well as onward HIV transmission. Regular HIV viral load testing is a key component for monitoring the effectiveness of ART in current WHO guidelines, which recently endorsed the use of point-of-care viral load assays, where testing is conducted at the clinical site by laboratory professionals or specifically-trained healthcare workers.^4^ These assays can reduce the time from blood draw to results being available, allowing clinicians to take decisions on the same day in one clinic visit, rather than having to schedule follow up appointments to discuss the results.^5,6^ A randomised trial conducted by our team in South Africa, using the Xpert HIV-1 VL assay(Cepheid, Sunnyvale, USA), found that point-of-care testing improved the primary outcome of HIV viral suppression and retention in care by 13.7%.^7^

Currently, there are two point-of-care HIV viral load assays that are approved by the WHO for use in low and middle-income countries. The m-PIMA HIV 1/2 VL (Abbott, Chicago, USA) received WHO pre-qualification in 2019,^8^ and has been found to perform well in one laboratory-based^9^ and two clinic-based evaluation studies.^10,11^ The Xpert HIV-1 VL received WHO pre-qualification in 2017,^12^ and has also been found to perform well in 19 studies, which have been summarised in two systematic reviews.^13,14^ Only three of these studies reported conducting Xpert HIV-1 VL testing in clinics, as opposed to centralised laboratories. Because the assay was not able to detect certain recombinant HIV-1 sub-types,^15^ Cepheid have recently released a next generation dual target assay, the Xpert HIV-1 Viral Load XC (HIV VL XC), that detects a wider range of HIV subtypes.^16^ This assay recently received CE-IVD clearance,^17^ but has not yet been evaluated in any published studies that we are aware of.

### Objectives

In this study, we aimed to assess the analytic performance of the novel, dual target Xpert HIV-1 VL XC, the existing Xpert HIV-1 VL and the m-PIMA HIV 1/2 VL, when used in South African clinics, compared to an established laboratory reference assay.

## Methods

### Study design and participants

We conducted a prospective diagnostic accuracy evaluation as a sub-study within the Point-Of-care HIV viral load testing to Enhance Resuppression (POwER) study, and the Centre for the AIDS Program of Research in South Africa (CAPRISA) 002 Acute Infection study. POwER is a randomised feasibility study in one urban and one rural public sector, primary care clinic in KwaZulu-Natal, South Africa, and the study protocol has been published elsewhere.^18^ In summary, approximately 100 non-pregnant adults aged 18 and over, receiving first-line ART and with HIV viraemia >1000 copies/mL in the past 6 weeks, are randomised to receive a second viral load test after 12 weeks by point-of-care test, or by standard laboratory based viral load testing. The primary outcome of viral suppression <50 copies/mL is assessed after 24 weeks. The CAPRISA 002 study is taking place in the same two clinics, and is a long-term cohort study of women living with HIV who are on ART.^19^ For this sub-study, POwER and CAPRISA 002 participants who provided written informed consent, had additional 4 to 8mLs of venous blood drawn for point-of-care assay evaluation, taken alongside the main study blood draws at enrolment or during follow up. We initially planned to use consecutive participants enrolling in POwER and attending follow up in CAPRISA 002, but disruptions in test cartridge supply chains, assay training and staffing due to the COVID-19 pandemic meant that there were periods where various assay cartridges were not available or rarely nurses did not have time to conduct testing. We therefore used a convenience sampling strategy instead, where consenting patients enrolling or attending follow up in either study had samples tested on whichever assays were available at that time in the respective clinic. This meant that some participants in POwER had multiple samples taken for this evaluation at up to three different visits.

### Test methods

Assay manufacturers provided point-of-care training to both research laboratory technicians and research nurses, as well as ongoing testing support. Initially, laboratory technicians conducted testing in a dedicated on-site point-of-care laboratory in the clinics. Once laboratory technician testing was established, research nurses conducted testing in a dedicated space in the clinic, with ongoing troubleshooting support from the manufacturer and on-site laboratory staff. For point-of-care viral load testing, 4 to 8mLs whole blood was obtained by venepuncture into an EDTA tube and centrifuged (by laboratory technicians or nurses respectively) to obtain plasma, which was transferred into the test cartridge and loaded into the respective assay platform. The m-PIMA HIV 1/2 platform is a single module instrument which requires 50 µL of plasma, and provides a quantitative viral load result between 800 to 1,000,000 copies/mL in approximately 70 minutes. The manufacturer states that the m-PIMA HIV 1/2 has a lower limit of detection of 342 (95% confidence interval [CI] 279 - 451) copies/mL for HIV-1 group M, and 228 (95% CI 187-295) copies/mL for HIV-1 group O.^20^ The new Xpert HIV 1 VL XC and existing Xpert HIV 1 VL use the same GeneXpert platform (Cepheid, Sunnyvale, USA) and both require 1 mL of plasma to provide a quantitative viral load result between 40 and 10,000,000 copies/mL in approximately 90 minutes. The lower limit of detection as stated by the manufacturer is 13.6 (95% CI 11.1-15.6) copies/mL for the Xpert HIV-1 VL XC,^16^ and 15.3 (95% CI 13.5-17.0) copies/mL for the Xpert HIV-1 VL.^21^ We used research use only cartridges of the same version as the now commercially released Xpert XC, and single module GXP I and four module GXP IV instruments. If a point-of-care assay resulted in an error or an invalid result, the test was repeated using remaining plasma, if available. Point-of-care viral load results from this evaluation were not provided to patients and were not used for clinical decision making.

For reference laboratory testing, a nurse drew whole blood in an EDTA tube at the same timepoint, which was centrifuged to plasma and stored at -80°C in the CAPRISA bio-repository. These samples were then defrosted and tested in two batches (study mid-point and study end) on the cobas^®^ HIV-1 reference assay using the cobas 6800 platform (Roche, Basel, Switzerland) at the Inkosi Albert Luthuli Hospital National Health Laboratory Service in Durban, KwaZulu-Natal. We chose this assay because it is one of the standard viral load assays which has been widely validated and used in the South African National Health Laboratory Service. Staff performing laboratory reference testing were blinded to results of the point-of-care testing and vice versa.

### Analysis

For the Xpert assays, we evaluated sensitivity and specificity to detect viraemia at thresholds of 50 copies/mL, 200 copies/mL and 1000 copies/mL. Because the m-PIMA HIV 1/2 VL does not provide a quantitative viral load below 800 copies/mL, we only evaluated the 1000 copies/mL threshold. We evaluated accuracy at these thresholds as they are used in national and international guidelines to define viraemia and treatment failure.^4,22–24^ We assessed agreement with the laboratory reference assay using Bland-Altman plots, and association using Pearson correlation coefficients, restricted to paired samples with a quantifiable viral load on both the point of care and reference laboratory assay.

We conducted sensitivity analyses using bootstrap methods (see supplementary material for methods) to account for repeat testing in the same participants at different time points, which could introduce clustering of errors. For example, if a participant had a viral sub-type that was not detected by a particular assay, inaccurate results would be more common among repeat tests in that participant, compared to tests in the general sample.

We estimated that with the resources and time available, we could test approximately 150 samples on each assay. However, the COVID-19 pandemic interrupted cartridge supply chains for the m-PIMA HIV 1/2 VL, meaning fewer tests were possible for this assay. Because not all samples were tested on all point-of-care assays, we did not directly compare the performance of the point-of-care assays against each other.

### Ethical approval

The POwER and CAPRISA 002 protocols, including use of samples for point-of-care viral load assay evaluation, were approved by the University of KwaZulu-Natal Biomedical Research Ethics Committee (BREC/00000836/2019 and E013/04 respectively), and POwER has also been approved by the University of Oxford Tropical Research Ethics Committee (OxTREC 66-19) and is registered with the Pan-African Clinical Trials Registry (PACTR202001785886049).

## Results

### Participants

Between June 26, 2020 and September 15, 2021, 140 participants provided paired samples, at one or more timepoints, for testing in this evaluation. Testing was conducted in the rural clinic for 25 participants, and at the urban clinic for 115 participants. 98 (70%) were women, median age was 37 years (interquartile range [IQR] 33-43), median time on ART was 4.6 years (IQR 2.9-6.0) and median CD4 count was 569 cells/µL (IQR 356-792).

### Test results

Overall, 189 samples were tested on at least one point-of-care viral load assay. 174 samples were tested using the Xpert HIV-1 VL XC, 188 samples using the Xpert HIV-1 VL, and 128 samples using the m-PIMA HIV 1/2 VL (Table 1). Nurses conducted testing for 54/174 (31%) of Xpert HIV-1 VL XC samples, 26/188 (14%) Xpert HIV-1 VL samples, and 23/128 (18%) m-PIMA HIV 1/2 VL samples. Initial errors were produced for eight (4.6%) Xpert HIV-1 VL XC samples, two (1.1%) Xpert HIV-1 VL samples, and three (2.5%) m-PIMA HIV 1/2 VL samples. In some cases, tests were successfully repeated, meaning that final results were not available due to assay errors for eight (4.6%) Xpert HIV-1 VL XC samples, one (0.5%) Xpert HIV-1 VL sample, and two (1.7%) m-PIMA HIV 1/2 VL samples (see Table 1 for total number of errors and Table S1 for error codes). Four of the Xpert HIV-1 VL XC errors occurred at the beginning of the nurse testing process; this was the first assay that the nurses were trained to use.

### Sensitivity and specificity

Sensitivity and specificity at 1000 copies/mL, compared to the Cobas 6800, were 97% and 98% (Xpert XC), 100% and 96% (Xpert VL), and 92% and 99% (m-PIMA) respectively. For the Xpert assays, sensitivity and specificity at 50 copies/mL were 93% and 96% (Xpert XC), and 95% and 95% (Xpert VL) respectively (Table 2). The m-PIMA was not assessed at 50 and 200 copies/mL because the lower limit of quantitation is 800 copies/mL.

### Correlation and agreement

**Fig 1:**
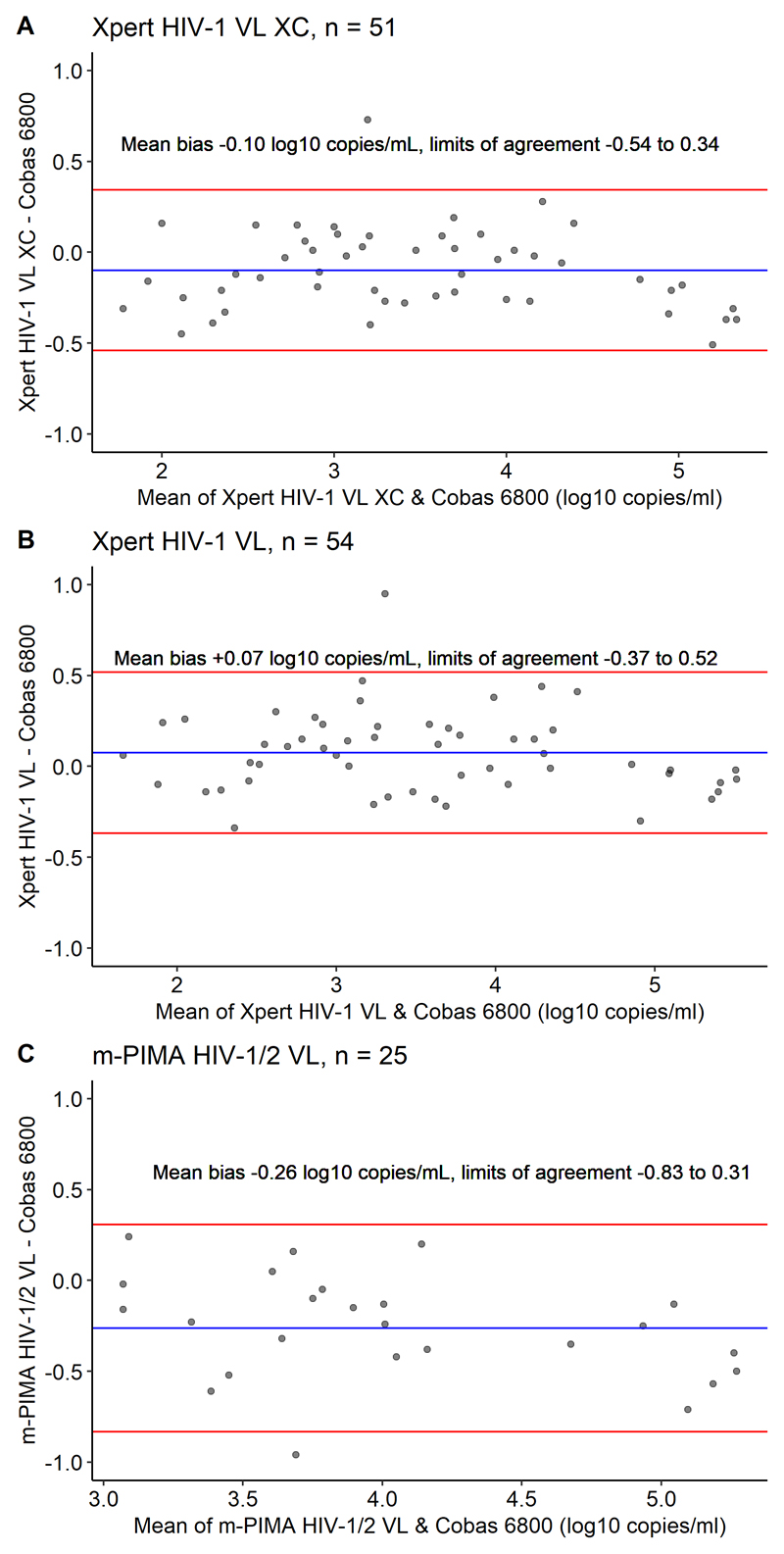
Bland Altman plots of Xpert HIV-1 VL XC, Xpert HIV-1 VL & m-PIMA HIV 1/2 vs Cobas 6800 Restricted to paired samples quantifiable on both the point-of-care and laboratory reference assay. The point estimate of the mean difference (bias) between the point-of-care and laboratory reference assay was within +/- 0.3 log_10_ copies/mL for all three assays.

**Fig 2:**
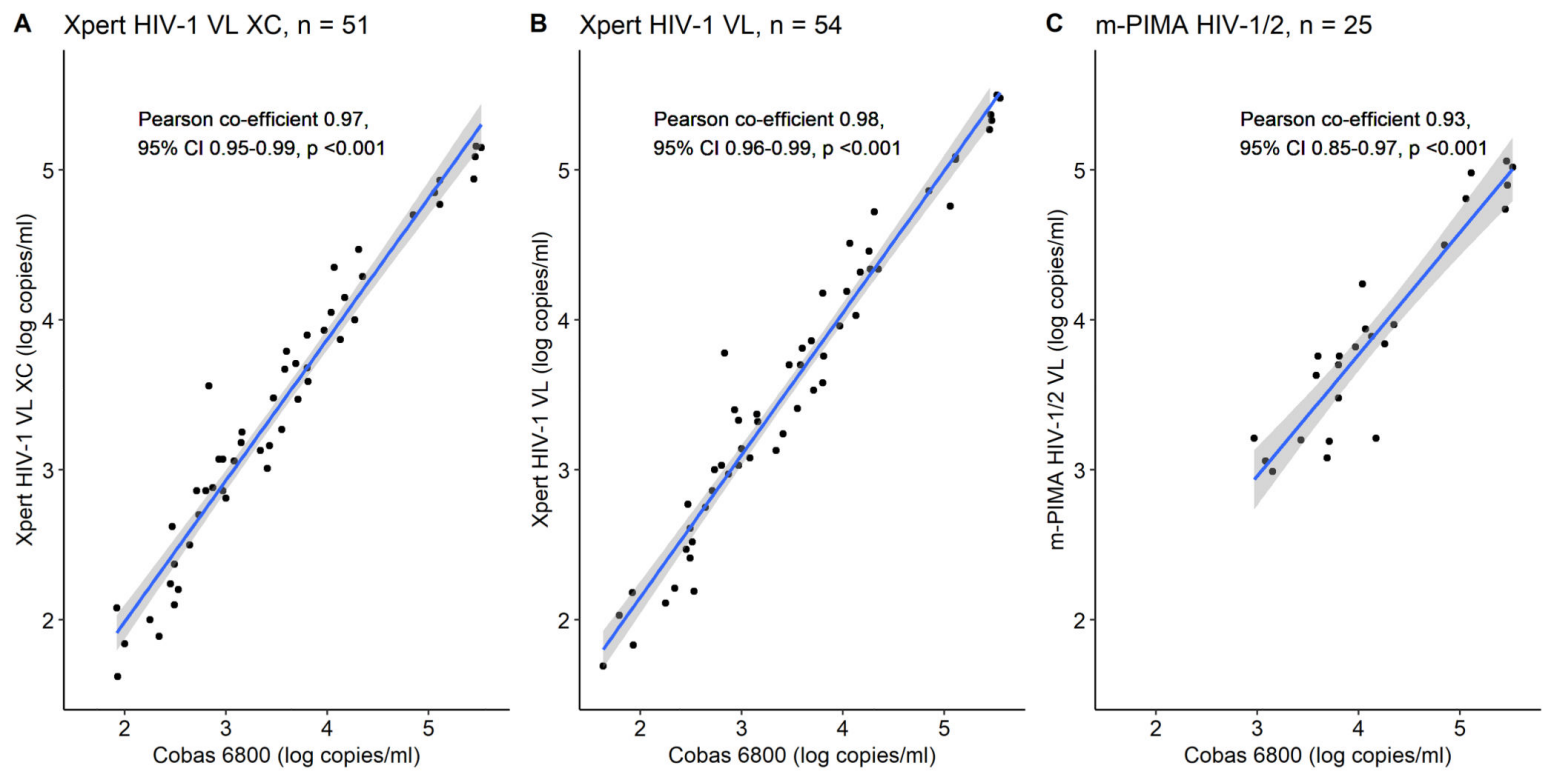
Correlation of Xpert HIV-1 VL XC, Xpert HIV-1 VL & m-PIMA HIV 1/2 VL vs Cobas 6800 Restricted to paired samples quantifiable on both the point-of-care and laboratory reference assay

When restricted to paired samples quantifiable at ≥40 copies/mL, the Xpert HIV-1 VL XC had a mean bias of -0.10 log_10_ copies/mL (95% limits of agreement -0.54 to 0.34,Fig 1A) and a Pearson correlation co-efficient of 0.97 (95% CI 0.95 to 0.99, p < 0.001, r^2^ = 0.95, Fig 2A), while the Xpert HIV-1 VL had a mean bias of +0.07 log_10_ copies/mL (95% limits of agreement -0.37 to 0.52, Fig 1B) and Pearson correlation co-efficient of 0.98 (95% CI 0.96-0.99, p <0.001, r^2^ = 0.95, Fig 2B). For the m-PIMA HIV 1/2 VL, when restricted to paired samples quantifiable at ≥800 copies/mL, the mean bias was -0.26 log_10_ copies/mL (95% limits of agreement -0.83 to 0.31, Fig 1C) and the Pearson correlation co-efficient was 0.93 (95% CI 0.85-0.97, p <0.001, r^2^ = 0.87, Fig 2C).

### Nurse-led testing

Accuracy, correlation and agreement of the assays were comparable when conducted by nurses and laboratory technicians, but numbers of nurse tested samples were small, particularly for the Xpert HIV-1 VL and the m-PIMA HIV-1/2 VL (Tables S2 and S3).

### Sensitivity analyses

In sensitivity analyses using bootstrap methods to account for some participants having samples drawn for evaluation at repeated timepoints, results were relatively similar (Table S4).

## Discussion

### Summary

We found that the novel Xpert HIV-1 VL XC, the Xpert HIV-1 VL and the m-PIMA HIV-1/2 VL had clinically acceptable performance when used in a South African clinic. At the WHO threshold for treatment failure of 1000 copies/mL, both the Xpert HIV-1 VL XC and Xpert HIV-1 VL had >95% sensitivity and specificity, while the m-PIMA HIV-1/2 VL had sensitivity of 92% and specificity of 99%. Both Xpert assays were also accurate at detecting low level viraemia.

#### Related literature

Our findings are similar to other studies of these three point-of-care assays, the majority of which have been conducted in centralised laboratory settings.

#### Xpert HIV-1 VL XC

Our study is the first to evaluate the Xpert HIV-1 VL XC in a clinical setting. In a centralised laboratory-based study, 513 fresh, frozen and diluted samples, were tested on the Xpert HIV-1 VL XC and compared with the Alinity m HIV-1 reference assay (Abbott, Chicago, USA).^25^ The Xpert HIV-1 VL XC had a mean bias of +0.10 log_10_ copies/mL (95% CI -0.54 to 0.35), compared to the reference assay, and r^2^ was 0.9788. Taken together, these results suggest that the Xpert HIV-1 VL XC performs well when used in centralised laboratory and clinic-based settings.

#### Xpert HIV-1 VL

For the Xpert HIV-1 VL, our findings were similar to a meta-analysis of 13 studies including 3790 patients,^14^ which found a mean bias of 0.04 (95% CI: 0.02-0.07) copies/mL, and r^2^ of 0.941 (P<0.001). Sensitivity and specificity was >95% at 200 and 1000 copies/mL, but was not evaluated at 50 copies/mL. Instead, a threshold of ‘detectable’ was used (40 copies/mL on the Xpert HIV-1 VL assay versus 20, 25 and 40 copies/mL in the different comparator assays used in the studies), with sensitivity of 93.3% (88.2–96.3), and much lower specificity at 80.6% (64.6–90.4). Specificity was particularly poor (69.3%) when compared to the Abbott RealTime HIV-1 VL assay. We used the 50 copies/mL threshold as this is used in South African guidelines.^22^ Our finding that both the Xpert HIV-1 VL XC and Xpert HIV-1 VL performed well at this threshold contrasts slightly with the systematic review findings, but suggests that these assays are appropriate for use in the South African setting.

#### m-PIMA HIV-1/2 VL

Three published studies have evaluated the m-PIMA HIV-1/2 VL assay. Two of these were clinic-based; one multi-site study in Mozambique compared 686 samples tested by nurses with the m-PIMA HIV-1/2 VL versus the Cobas AmpliPrep/Cobas TaqMan 96 HIV-1 v2 (Roche) assay,^10^ while another multi-site study from Kenya compared 555 samples tested by laboratory technicians on the m-PIMA HIV-1/2 VL in clinics, with the RealTime HIV-1 assay (Abbott).^11^ The final study was a centralised laboratory evaluation comparing 413 frozen samples tested on the m-PIMA HIV -1/2 VL with results from fresh samples tested on the RealTime HIV-1 assay. At 1000 copies/mL, sensitivity and specificity were above 95% in all studies, except for the laboratory evaluation where specificity was 76.9 % (95% CI: 69.8–83.1). The correlation coefficients were 0.92, 0.92 and 0.93 respectively, with mean bias of -0.20, +0.16 and +0.26 log_10_ copies/mL respectively versus the laboratory reference assay. The WHO prequalification report found a bias of -0.21 log_10_ copies/mL versus the COBAS AmpliPrep/COBAS TaqMan HIV-1 Test, v2.0, with sensitivity of 95.1% and specificity of 99% at 1000 copies/mL^8^. Overall, these findings, particularly when using Roche reference laboratory assays, are similar to our results, although reasons for the low specificity in the laboratory study are not clear, but could be related to the use of frozen samples for m-PIMA testing.

#### Nurse testing

There is only one published study which has evaluated nurse-led quantitative point-of-care viral load testing, with good results using the m-PIMA HIV1/2 viral load in Mozambique, as described above.^10^ Our data suggest that the Xpert HIV-1 VL XC performs well when used by nurses, and results with the Xpert HIV-1 VL and m-PIMA HIV-1/2 VL were relatively similar, although numbers were small. However, our study was not explicitly designed to compare nurse versus laboratory testing, and research nurses had support available to them from on-site laboratory technicians, which does not reflect conditions across South African primary care. Nurses and/or midwives have accurately used qualitative viral load assays (that use the GeneXpert and m-PIMA platforms) for early infant diagnosis of HIV,^26,27^ and there is increasing interest in task-shifting point-of-care HIV diagnostics to non-laboratory staff in low and middle income countries.^28–30^ As these assays require plasma, venous blood samples had to be centrifuged by nurses in the study clinic. This required specific training and may not be feasible for nurses in many settings, which could limit use at the clinical point-of-care. A whole blood fingerstick assay would circumvent this problem, and point-of-care early infant diagnosis assays, which also use whole blood, have demonstrated clear benefits in clinical outcomes.^26,31^ This highlights the potential benefits of providing same day results using point-of-care technologies.

### Strengths and limitations

Strengths of our study include the assessment of clinic-based testing in a setting with high HIV prevalence, and the inclusion of multiple point-of-care assays. As well as assessing correlation and mean bias, we focussed our analysis on sensitivity and specificity to detect viraemia at viral load thresholds that are directly relevant for patient management in South Africa and internationally. Most viral load testing was conducted by laboratory technicians, but we included some nurse led viral load testing, which has not been widely evaluated previously. Supply chain and operational disruptions due to the COVID-19 pandemic meant that our sample size was smaller than anticipated for some assays, and we were unable to test samples on all three assays concurrently. Instead, we allowed paired samples from repeat timepoints in the same participant, and conducted sensitivity analyses to account for potential clustering, with similar results. Nevertheless, the sample size of this study is relatively small, meaning confidence intervals for sensitivity estimates are wide for all assays at all viraemia thresholds, and for sensitivity and specificity estimates for nurse testing. Reference laboratory testing was conducted retrospectively using frozen plasma samples, in accordance with manufacturer’s instructions, which should not have affected results.^32^

### Implications

A previous cost-effectiveness modelling study found that a sensitivity and specificity of 90% or more at a threshold of 1000 copies/mL would be acceptable for a point-of-care viral load assay in Southern Africa,^33^ while mean bias should ideally be within 0.3 log_10_ copies/mL of reference laboratory assays.^6,34^ In our evaluation, all three evaluated assays fell within these criteria.

Balancing assay analytic performance and other characteristics that influence clinical outcomes, such as ease of use, turn-around time and result availability will depend on the context of where the assay is being implemented. We found that the Xpert HIV-1 VL XC performed well at both the 50 copies/mL and 1000 copies/mL thresholds, which are the two key thresholds for ART management in South Africa, suggesting that the clinical performance is appropriate for this setting. The m-PIMA HIV-1/2 VL is not able to quantitate viral loads <800 copies/mL, making it less suited to assessment of low level viraemia. More recent evidence has found that low level viraemia among people receiving efavirenz-based ART regimens predicts subsequent treatment failure,^35^ although whether this remains true on dolutegravir based regimens is not yet known.^36^ South African guidelines currently use 50 copies/mL to define viraemia, and determine whether a patient is eligible for down-referral into streamlined ART delivery programmes,^37,38^ or whether they need enhanced adherence counselling to encourage resuppression,^37^ meaning the m-PIMA is less suited for use in these situations. However, the m-PIMA has a simple interface, shorter run time, and may be more suited to implementation in clinic-based settings, particularly in settings where access to viral load testing is poor. Further work is needed to confirm the performance of these assays when used by nurses, and we are conducting qualitative work with nurses and other healthcare workers, to explore feasibility and acceptability of implementing nurse testing in clinical settings. Using these assays to provide same-day, point-of-care results is crucial to achieving the reported benefits of expedited clinical decision-making, and reduced clinic visits and patient travel costs.^39^

### Conclusions

The diagnostic accuracy of the Xpert HIV-1 VL XC, Xpert HIV-1 VL and m-PIMA HIV-1/2 VL assays was clinically acceptable when testing was conducted in these South African clinics. The Xpert assays have the added benefit of accurately detecting low level viraemia. However, further clinical studies are needed to determine if these assays can be implemented in routine primary healthcare settings, and whether there are operational and clinical benefits to patients and providers when used at the clinical point-of-care.

## Supplementary Material

Supplementary material

## Figures and Tables

**Table 1:** Samples tested on point-of-care viral load assays

	Xpert HIV-1 VL XC	Xpert HIV-1 VL	m-PIMA HIV 1/2 VL
Samples from one timepoint per participant	84 (48.3)	97 (51.6)	67 (56.8)
Samples from two timepoints per participant	70 (40.2)	78 (41.5)	46 (39.0)
Samples from 3 timepoints per participant	12 (6.9)	12 (6.4)	3 (2.5)
No result due to assay errors ^a^	8 (4.6) ^b^	1 (0.5) ^c^	2 (1.7) ^d^
Total	174	188	118

a See Table S1 for error codes

b Eight samples (4.6%) produced an error. Four were retested, with the repeat test also producing an error meaning no result was available. Four were not retested meaning no result was available

c Two samples (1.1%) produced an error, and both were retested. One repeat test also produced an error meaning no result was available, the other produced a valid result.

d Three samples (2.5%) produced an error, and all were retested. Two repeat tests also produced an error meaning no result was available, the other produced a valid result.

**Table 2:** Raw numbers, sensitivity, and specificity (95% confidence intervals) of three point-of-care viral load assays at different viral load thresholds

		Roche Cobas 6800					Roche Cobas 6800					Roche Cobas 6800		
		*<50*	*≥50*	*Total*			*<50*	*≥50*	*Total*			*<50*	*≥50*	*Total*
**Xpert HIV-1 VL XC**	*<50*	108	4	112	**Xpert HIV-1 VL**	*<50*	125	3	128	**m-PIMA HIV 1/2 VL**	*<50*	-	-	-
	*≥50*	4	50	54		*≥50*	6	53	59		*≥50*	-	-	-
	*Total*	112	54	166		*Total*	131	56	187		*Total*	-	-	-
	Sensitivity: 0.93 (0.81, 0.98) Specificity: 0.96 (0.91, 0.99)					Sensitivity: 0.95 (0.84, 0.99) Specificity: 0.95 (0.90, 0.98)					Sensitivity: NA Specificity: NA			
		*<200*	*≥200*	*Total*			*<200*	*≥200*	*Total*			*<200*	*≥200*	*Total*
**Xpert HIV-1 VL XC**	*<200*	116	4	120	**Xpert HIV-1 VL**	*<200*	137	2	139	**m-PIMA HIV 1/2 VL**	*<200*	-	-	-
	*≥200*	3	43	46		*≥200*	1	47	48		*≥200*	-	-	-
	*Total*	119	47	166		*Total*	138	49	187		*Total*	-	-	-
	Sensitivity: 0.91 (0.79, 0.97) Specificity: 0.97 (0.92, 0.99)					Sensitivity: 0.96 (0.85, 0.99) Specificity: 0.99 (0.95, 1.00)					Sensitivity: NA Specificity: NA			
		*<1000*	*≥1000*	*Total*			*<1000*	*≥1000*	*Total*			*<1000*	*≥1000*	*Total*
**Xpert HIV-1 VL XC**	*<1000*	131	1	132	**Xpert HIV-1 VL**	*<1000*	148	0	148	**m-PIMA HIV 1/2 VL**	*<1000*	90	2	92
	*≥1000*	3	31	34		*≥1000*	6	33	39		*≥1000*	1	23	24
	*Total*	134	32	166		*Total*	154	33	187		*Total*	91	25	116
	Sensitivity: 0.97 (0.82, 1.00) Specificity: 0.98 (0.93, 0.99)					Sensitivity: 1.00 (0.87, 1.00) Specificity: 0.96 (0.91, 0.98)					Sensitivity: 0.92 (0.72-0.99) Specificity: 0.99 (0.93-1.00)			
