# Supplementary material for: Diagnostic accuracy of the rapid Xpert HIV-1 Viral Load XC, Xpert HIV-1 Viral Load & m-PIMA HIV-1/2 Viral Load in South African clinics
Source: J Acquir Immune Defic Syndr. Author manuscript; Available in PMC 2022 Oct 1. (PMC7613592; doi:10.1097/QAI.0000000000003037)
Supplement: Supplementary material [file EMS145976-supplement-Supplementary_material.docx]

**SUPPLEMENTARY MATERIAL**

**Contents**

[Table S1: Error codes for invalid point-of-care viral load assay results 1](#_Toc103933978)

[Table S2: Sensitivity and specificity of three point-of-care viral load assays when used by laboratory technicians and nurses 4](#_Toc103933979)

[Table S3: Mean log_10_ bias and correlation coefficients of three point-of-care viral load assays when used by laboratory technicians and nurses 4](#_Toc103933980)

[Table S4: Bootstrap estimates of sensitivity, specificity, bias and correlation of three point-of-care viral load assays at different viral load thresholds, taking into account repeat testing 5](#_Toc103933981)

## Table S1: Error codes for invalid point-of-care viral load assay results

| **ID** | **Tester** | **Error**  **code** | **Potential causes** | **Comment** |
| --- | --- | --- | --- | --- |
| **XPERT HIV-1 VL XC** | | | | |
| 8 | Lab technician | 5007 | Sample related: PCR inhibitors in sample  Instrument related: Sub-optimal optical channels | First test error |
| 8 | Lab technician | 5007 | Sample related: PCR inhibitors in sample  Instrument related: Sub-optimal optical channels | Repeat test error: no valid result |
| 9 | Lab technician | 5007 | Sample related: PCR inhibitors in sample  Instrument related: Sub-optimal optical channels | First test error |
| 9 | Lab technician | 5007 | Sample related: PCR inhibitors in sample  Instrument related: Sub-optimal optical channels | Repeat test error: no valid result |
| 33 | Lab technician | 2126 | Instrument related: Communication error, e.g. cables not properly connected | First test error |
| 33 | Lab technician | 2126 | Instrument related: Communication error, e.g. cables not properly connected | Repeat test error: no valid result |
| 34 | Lab technician | 2126 | Instrument related: Communication error, e.g. cables not properly connected | First test error |
| 34 | Lab technician | 2126 | Instrument related: Communication error, e.g. cables not properly connected | Repeat test error: no valid result |
| 79 | Nurse | 2126 | Instrument related: Communication error, e.g. cables not properly connected | First test error, no repeat so no valid result |
| 81 | Nurse | 2008 | Sample related: Pressure error most often due to viscosity or particulate matter | First test error, no repeat so no valid result |
| 54 | Nurse | 2097 | User related: Insufficient plasma volume | First test error, no repeat so no valid result |
| 89 | Nurse | 2096 | User related: Insufficient plasma volume | First test error, no repeat so no valid result |
| **XPERT HIV-1 VL** | | | | |
| 58 | Lab technician | 5007 | Sample related: PCR inhibitors in sample  Instrument related: Sub-optimal optical channels | Test repeated and valid result obtained |
| 85 | Nurse | 2037 | Cartridge-related: Pressure error | First test error |
| 85 | Nurse | 2097 | User related (insufficient plasma volume) | Repeat test error: no valid result |
| **MPIMA HIV-1/2 VL** | | | | |
| 437 | Lab technician | 23215 | Instrument related: lint / particle on optic | Result obtained on repeat |
| 402 | Lab technician | 23020 | Sample related: Interfering substances or coagulated  Cartridge related (seldom): Cartridge defect  Instrument related: Lysis heater or mixer module failure | First test error |
| 402 | Lab technician | 23020 | Sample related: Interfering substances or coagulated  Cartridge related (seldom): Cartridge defect  Instrument related: Lysis heater or mixer module failure | Repeat test error: no valid result |
| 92 | Lab technician | 23210 | User related: Incorrect cartridge insertion  Cartridge related: Microarray dislocated  Instrument related: Cartridge lock issue | First test error |
| 92 | Lab technician | 23210 | User related: Incorrect cartridge insertion  Cartridge related: Microarray dislocated  Instrument related: Cartridge lock issue | Repeat test error: no valid result |

## Table S2: Sensitivity and specificity of three point-of-care viral load assays when used by laboratory technicians and nurses

| **Assay** | **Threshold (copies/mL)** | **Laboratory technician testing** | | **Nurse testing** | |
| --- | --- | --- | --- | --- | --- |
|  |  | **Sensitivity (95% CI)** | **Specificity (95% CI)** | **Sensitivity (95% CI)** | **Specificity (95% CI)** |
| Xpert HIV-1 VL XC | N | 116 | | 50 | |
|  | 50 | 0.90 (0.73 to 0.97) | 0.98 (0.91 to 1.00) | 0.96 (0.76 to 1.00) | 0.93 (0.74 to 0.99) |
|  | 200 | 0.93 (0.74 to 0.99) | 0.99 (0.93 to 1.00) | 0.90 (0.67 to 0.98) | 0.93 (0.76 to 0.99) |
|  | 1000 | 0.95 (0.72 to 1.00) | 0.98 (0.92 to 1.00) | 1.00 (0.72 to 1.00) | 0.97 (0.84 to 1.00) |
| Xpert HIV-1 VL | N | 162 | | 25 | |
|  | 50 | 0.95 (0.82 to 0.99) | 0.95 (0.89 to 0.98) | 0.94 (0.68 to 1.00) | 1.00 (0.63 to 1.00) |
|  | 200 | 0.97 (0.84 to 1.00) | 0.99 (0.95 to 1.00) | 0.92 (0.62 to 1.00) | 1.00 (0.70 to 1.00) |
|  | 1000 | 1.00 (0.83 to 1.00) | 0.97 (0.92 to 0.99) | 1.00 (0.60 to 1.00) | 0.88 (0.62 to 0.98) |
| m-PIMA HIV-1/2 VL | N | 93 | | 23 | |
|  | 1000 | 0.94 (0.71 to 1.00) | 0.99 (0.92 to 1.00) | 0.86 (0.42 to 0.99) | 1.00 (0.76 to 1.00) |

## Table S3: Mean log_10_ bias and correlation coefficients of three point-of-care viral load assays when used by laboratory technicians and nurses

| **Assay** | **Laboratory technician testing** | | **Nurse testing** | |
| --- | --- | --- | --- | --- |
|  | **Mean bias, log_10_ copies/mL (95% CI)** | **Correlation co-efficient (95% CI)** | **Mean bias, log_10_ copies/mL (95% CI)** | **Correlation co-efficient (95% CI)** |
| N | 28 | | 23 | |
| Xpert HIV-1 VL XC (n = 123) | -0.12 (-0.49 to 0.24) | 0.98 (0.95 to 0.99) | -0.07 (-0.59 to 0.45) | 0.97 (0.93 to 0.99) |
| N | 38 | | 16 | |
| Xpert HIV-1 VL  (n = 140) | 0.04 (-0.34 to 0.41) | 0.98 (0.96 to 1.00) | 0.16 (-0.39 to 0.71) | 0.98 (0.94 to 0.99) |
| N | 18 | | 7 | |
| m-PIMA HIV-1/2 VL  (n = 91) | -0.21 (-0.74 to 0.33) | 0.94 (0.80 to 0.97) | -0.40 (-1.01 to 0.21) | 0.93 (0.61 to 0.99) |

## Table S4: Bootstrap estimates of sensitivity, specificity, bias and correlation of three point-of-care viral load assays at different viral load thresholds, taking into account repeat testing

| **Assay** | **Threshold (copies/mL)** | **Sensitivity (95% CI)** | **Specificity (95% CI)** | **Mean bias, log_10_ copies/mL (95% CI)** | **Correlation co-efficient (95% CI)** |
| --- | --- | --- | --- | --- | --- |
| Xpert HIV-1 VL XC (n = 123) | 50 | 0.95 (0.81 to 0.99) | 0.98 (0.92 to 1.00) | -0.10 (-0.57 to 0.38) | 0.97 (0.94 to 0.99) |
|  | 200 | 0.89 (0.73 to 0.97) | 0.99 (0.93 to 1.00) |  |  |
|  | 1000 | 0.98 (0.79 to 1.00) | 0.98 (0.92 to 0.99) |  |  |
| Xpert HIV-1 VL  (n = 140) | 50 | 0.95 (0.81 to 0.99) | 0.96 (0.90 to 0.99) | 0.07 (-0.37 to 0.52) | 0.97 (0.95 to 0.99) |
|  | 200 | 0.96 (0.81 to 0.99) | 0.99 (0.95 to 1.00) |  |  |
|  | 1000 | 1.00 (0.82 to 1.00) | 0.97 (0.91 to 0.99) |  |  |
| m-PIMA HIV-1/2 VL  (n = 91) | 1000 | 0.89 (0.63 to 0.98) | 0.99 (0.93 to 1.00) | -0.30 (-0.89 to 0.30) | 0.93 (0.80 to 0.97) |

In the bootstrap models we randomly selected samples from one timepoint per participant, and calculated the sensitivity, specificity, mean bias and correlation from this population. We then repeated this 1000 times, and calculated the mean sensitivity, specificity, mean bias and correlation.
